# Genetic Evidence Supporting the Role of the Calcium Channel, CACNA1S, in Tooth Cusp and Root Patterning

**DOI:** 10.3389/fphys.2018.01329

**Published:** 2018-09-26

**Authors:** Virginie Laugel-Haushalter, Supawich Morkmued, Corinne Stoetzel, Véronique Geoffroy, Jean Muller, Anne Boland, Jean-François Deleuze, Kirsley Chennen, Waranuch Pitiphat, Hélène Dollfus, Karen Niederreither, Agnès Bloch-Zupan, Patimaporn Pungchanchaikul

**Affiliations:** ^1^Laboratoire de Génétique Médicale, UMR_S INSERM U1112, IGMA, Faculté de Médecine, FMTS, Université de Strasbourg, Strasbourg, France; ^2^Biofilm Research Group, Department of Pediatric Dentistry, Faculty of Dentistry, Khon Kaen University, Khon Kaen, Thailand; ^3^Institut de Génétique et de Biologie Moléculaire et Cellulaire (IGBMC), CERBM, INSERM U 1258, CNRS- UMR 7104, Université de Strasbourg, Strasbourg, France; ^4^Laboratoires de Diagnostic Génétique, Hôpitaux Universitaires de Strasbourg, Strasbourg, France; ^5^Centre National de Recherche en Génomique Humaine (CNRGH), Institut de Biologie François Jacob, Direction de la Recherche Fondamentale, Commissariat à l’Energie Atomique et aux Energies Alternatives, Paris, France; ^6^Department of Computer Science, ICube, CNRS - UMR 7357, Fédération de Médecine Translationnelle de Strasbourg, Université de Strasbourg, Strasbourg, France; ^7^Department of Community Dentistry, Faculty of Dentistry, Khon Kaen University, Khon Kaen, Thailand; ^8^Centre de Référence pour les Affections Rares en Génétique Ophtalmologique, Filière SENSGENE, Hôpitaux Universitaires de Strasbourg, Strasbourg, France; ^9^Faculté de Chirurgie Dentaire, Université de Strasbourg, Strasbourg, France; ^10^Hôpitaux Universitaires de Strasbourg (HUS), Pôle de Médecine et Chirurgie Bucco-Dentaires Hôpital Civil, Centre de Référence des Maladies Rares Orales et Dentaires, O-Rares, Filière Santé Maladies Rares TETE COU, European Reference Network ERN CRANIO, Strasbourg, France

**Keywords:** rare disease, dental anomalies, patterning, mutations, NGS, human, calcium ion channel

## Abstract

In this study, we report a unique dominantly inherited disorganized supernumerary cusp and single root phenotype presented by 11 affected individuals belonging to 5 north-eastern Thai families. Using whole exome sequencing (WES) we identified a common single missense mutation that segregates with the phenotype in exon 6 of *CACNA1S* (Ca_v_1.1) (NM_000069.2: c.[865A > G];[=] p.[Ile289Val];[=]), the Calcium Channel, Voltage-Dependent, L Type, Alpha-1s Subunit, OMIM ^∗^ 114208), affecting a highly conserved amino-acid isoleucine residue within the pore forming subdomain of CACNA1S protein. This is a strong genetic evidence that a voltage-dependent calcium ion channel is likely to play a role in influencing tooth morphogenesis and patterning.

## Introduction

Advances in molecular biology have increased our in-depth understanding of major processes directing tooth morphogenesis (Jernvall and Thesleff, 2012). Odontogenesis occurs in sequential developmental stages initiated with dental epithelial placode induction (seen as localized thickening of the oral ectoderm), followed by the bud, cap, and bell morphogenetic stages. This is followed by the terminal differentiations of odontoblasts and ameloblasts. Subsequently, root formation and tooth eruption lead to the formation of sub-regional tooth types (incisors, canines, premolars and molars), which are distinguished by a unique shape and size of both the crown and root. This regionalized layout is defined with single cusps and roots present in incisors and canines and multiple cusps and roots in molars; premolars display an intermediate crown and root pattern (2–3 cusps and single to double roots). Studies of developing mouse teeth suggest that both cusp and root initiation and patterning repeatedly re-utilize conserved developmental pathways participating in interactions between the oral ectoderm and cephalic neural crest-derived ectomesenchymal cells (Thesleff and Mikkola, 2014; Balic and Thesleff, 2015).

During dental development, the number of cusps is controlled by enamel knots, which are morphogen-expressing signaling centers (Matalova et al., 2005; Ahtiainen et al., 2016). Indeed cusps form precisely at the location of secondary enamel knot signaling centers, which function as trophic regions inducing cusp growth and controlling the fine definition of cusp form (Jernvall et al., 2000). To date, molecular mechanisms regulating enamel knot development are understood in the overall context of dental morphogenesis. Cusp number regulation stems from primary patterning events relying on multiple known dental regulatory pathways such as sonic hedgehog, fibroblast growth factor, and ectodysplasin (Bei, 2009; Charles et al., 2009a,b Harjunmaa et al., 2012). Rare syndromic and non-syndromic alterations affecting human tooth development offer opportunities to understand unique mechanisms regulating tooth number or cusp morphology. To date, heritable genetic alterations increasing cusp number are relatively rare in human populations. In this study, we explore a dominantly inherited supernumerary cusp phenotype in 11 affected individuals belonging to 5 Thai families. Using next generation sequencing (NGS) we identified a single shared variant, a missense mutation in *CACNA1S*, the gene encoding the Calcium Channel, Voltage-Dependent, L Type, Alpha-1s Subunit, CaV.1 (1q32.1, OMIM × 114208), affecting a highly conserved amino-acid isoleucine residue within the pore forming subdomain of CACNA1S protein and segregating with the phenotype. This is a strong genetic evidence that a voltage-dependent calcium ion channel is likely to play a role in influencing tooth morphogenesis and patterning.

## Materials and Methods

### Patients

The patients and their families were examined at Khon Kaen University Faculty of Dentistry and Hospital in Thailand. Affected (11), non-affected (4), as well as unrelated control individuals (18) consented to participate in the study named “Identification of the gene involved in a unique dental anomaly phenotype: multiple tuberculated permanent teeth encountered in Thai families.” They were recruited between 2012 and 2017. This study ST 0514.13.7/7241, Reference No. HE542262, and accompanying documents (information, consent forms) were reviewed and approved in 2012 by the Khon Kaen University Ethics Committee for Human Research based on the Declaration of Helsinki and ICH Good Clinical Practice Guidelines.

**FIGURE 1:**
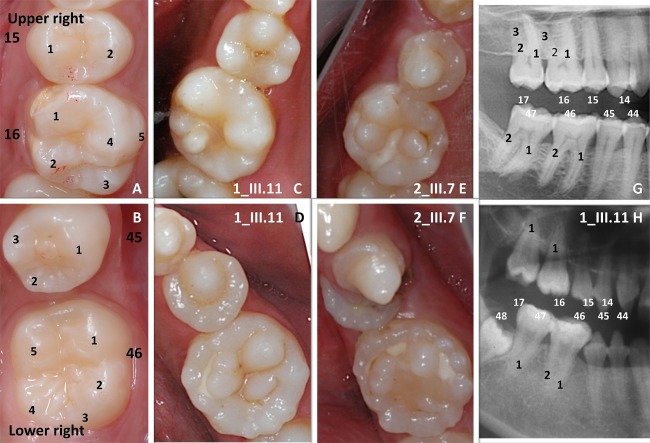
A striking multiple cusps single root phenotype. Normal crown morphology **(A,B)** of the right upper second premolar (15) and first permanent molar (16) and of the right lower premolar (45) and first permanent molar (46) could be compared to the multicusp pattern phenotype clearly visible both in the upper (**C** 1_III.11, **E** 2_III.7) and lower (**D** 1_III.11, **F** 2_III.7) jaws. On the close up of the right premolar-molar area on panoramic radiographs (**G** control, **H** 1_III.11 affected) molars present a single root (1) pattern. Only the first lower permanent molar (46) shows a more apical root furcation leading to the taurodontic appearance of the tooth.

The oral phenotypes (**Figure 1**) were documented using the D[4]/phenodent registry, a Diagnosing Dental Defects Database^1^, which is approved by CNIL (French National Commission for Informatics and Liberty, number 908416). This clinical study is registered at https://clinicaltrials.gov: NCT01746121 and NCT02397824, and with the MESR (French Ministry of Higher Education and Research) Bioethics Commission as a biological collection “Orodental Manifestations of Rare Diseases” DC-2012-1677 within DC-2012-1002 and was acknowledged by the CPP (person protection committee) Est IV December 11th 2012.

Affected and non-affected family members and enrolled subjects gave written informed consents in accordance with the Declaration of Helsinki, both for the D[4]/phenodent registry and for genetic analyses performed on the salivary samples included in the biological collection. A material transfer agreement (MTA) was signed between collaborating universities and laboratories.

### Sample Collection

**FIGURE 2:**
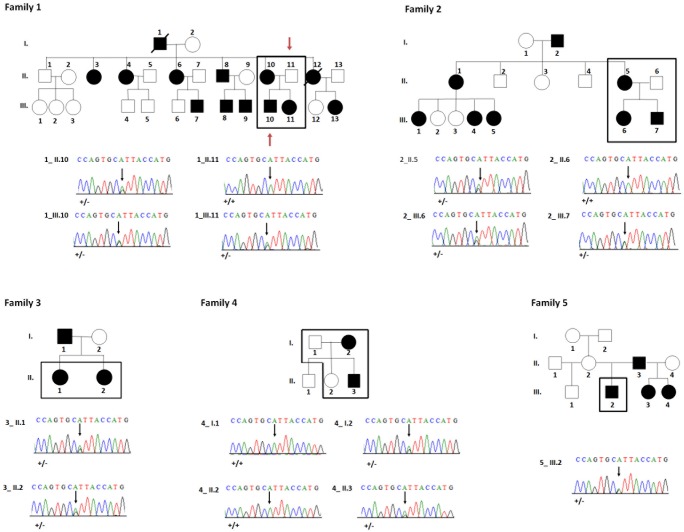
Pedigrees and sequences analysis of families 1–5. Black squares indicate the patients analyzed in this study. All available members from families 2 and 3 were analyzed by whole exome sequencing. In family 1 only the 2 individuals [indicated by a red arrow (1_II.11, 1_III.10) were analyzed by WES]. Families 4 and 5 were analyzed exclusively by Sanger sequencing. Electropherograms reveal a heterozygous missense mutation c.865A > G; p.Ile289Val in affected individuals (1_II.10, 1_III.10, 1_III.11, 2_II.5, 2_III.6, 2_III.7, 3_II.1, 3_II.2, 4_I.2, 4_II.3, 5_III.2).

Genomic DNA was isolated from the saliva of patients, unaffected family members, as well as control unrelated individuals using the Oragene^®^ DNA OG-510 or OG-250 commercial kits (DNA Genotek Inc., Kanata, ON, Canada ^2^) according to the manufacturer’s protocol. Whole exome sequencing (WES) was performed on eight individuals from families 1, 2, and 3, and Sanger sequencing was performed on exon 6 of the *CACNA1S* gene on all enrolled members of the five families (**Figure 2**), as well as on 18 unrelated and unaffected individuals from the same geographic region to serve, in combination with data provided by a Thaï^3^ and an Asian database (Ngamphiw et al., 2011), as single nucleotide polymorphism (SNP) controls.

### Whole-Exome Sequencing

Whole exome sequencing (WES) was performed at the Centre National de Recherche en Génomique Humaine (CNRGH, Institut de Biologie François Jacob, CEA, Evry, France). For family 1 only the father and the son were sequenced, (1_II.11 and 1_III.10), for family 2 all the available members were sequenced (2_II.5, 2_II.6, 2_III.6, and 2_III.7) and for family 3 only the two daughters were sequenced (3_II.1 and 3_II.2).

After quality control, genomic DNA (3 μg) was captured using the in-solution enrichment methodology (Human All Exon v5 – 50 Mb, Agilent Technologies, CA, United States). Library preparation and exome enrichment protocol (∼20.000 targeted genes) have been performed using an automated platform, according to manufacturer’s instructions (SureSelect, Agilent Technologies, CA, United States). After normalization and quality control, exome enriched libraries were sequenced on a HiSeq 2000 from Illumina (Illumina Inc., CA, United States) as paired-end 100 bp reads. Image analysis and base calling were performed using the Illumina Real-Time Analysis (RTA) Pipeline. Sequence quality parameters were assessed throughout the sequencing run. The standard bioinformatics analysis of sequencing data was based on the Illumina pipeline to generate FASTQ files for each sample. Sequence data was then processed using the exome analysis platform developed at CNRGH, which follows GATK best practice. Coverage/depth statistics have been accessed as quality control criteria. The sequencing yield generated for each sample allowed a minimum of 10× coverage for 93% of the targets. An average sequencing depth of at least 60× was obtained for good quality sample DNA. Polymorphism detection for each sample was performed using reads mapping procedure onto the reference genome (hg19) followed by “SNP calling” algorithm implemented by GATK/SAMtools software. Results (FASTQ, BAM and non-annotated VCF files) were communicated through a dedicated secure web site at CNRGH.

### Bioinformatics Analysis

Annotation, ranking and filtering of genetic variants were performed with the VaRank program (Geoffroy et al., 2015) in combination with the Alamut Batch software (Interactive Biosoftware, Rouen, France). Very stringent criteria were used for excluding non-pathogenic variants, in particular: (1) variants represented with an allele frequency of more than 1% in dbSNP 138 (Sherry et al., 2001), in the EXAC database (Karczewski et al., 2017), in the NHLBI Exome Sequencing Project Exome Variant Server (EVS) (NHLBI GO Exome Sequencing Project^4^) or in our internal exome database, (2) variants in the 5′ or 3′ UTR, (3) variants with intronic locations and no prediction of local splice effect, and (4) synonymous variants without prediction of local splice effect. Variant effect on the nearest splice site was predicted using MaxEntScan (Yeo and Burge, 2004), NNSplice (Reese et al., 1997) and Splice Site Finder (Shapiro and Senapathy, 1987). Our analysis focused on heterozygous variants (SNV/indel) shared by all affected individuals, consistent with a dominant mode of inheritance.

Structural variants were predicted using by default the CANOES program (Backenroth et al., 2014) and annotated thanks to our in-house script AnnotSV (Geoffroy et al., 2018) based on the classical annotations such as the Database of Genomic Variants (DGV).

For the evaluation of possible relationships between the eight sequenced individuals from families 1, 2, and 3 a multi-sample VCF was created. The generated multi-sample VCF was converted into a PLINK binary format and used by the KING program (v2.1.4) to estimate the pair-wise kinship coefficients (–kinship parameter) (Manichaikul et al., 2010). Close relatives were inferred based on the estimated kinship coefficients ranges >0.354, [0.177, 0.354], [0.0884, 0.177], and [0.0442, 0.0884] corresponding to duplicate/MZ twin, 1st-degree, 2nd-degree, and 3rd-degree relationships, respectively.

### Sanger Sequencing and Segregation

Primer pair for exon 6 of the *CACNA1S* gene was designed using Primer 3 (sense: 5′-GACATAATTCCCGCTGCCTG; antisense: 5′- GTTTCCATTCTTCACCCGCC). The amplification of the specific region of interest was performed on 50 ng of genomic DNA template. The PCR product was then purified and bidirectional Sanger sequencing performed by GATC Sequencing Facilities (Konstanz, Germany). All enrolled members of the five families and control subjects were assessed by Sanger sequencing. For family 1 (1_II.10 and 1_III.11), family 4 (4 individuals including an affected mother and son, 4_I.2 and 4_II.3, respectively) and family 5 (male 5_III.2) only Sanger sequencing and not exome sequencing was performed.

### Mutations in the Human *CACNA1S* Gene

**FIGURE 3:**
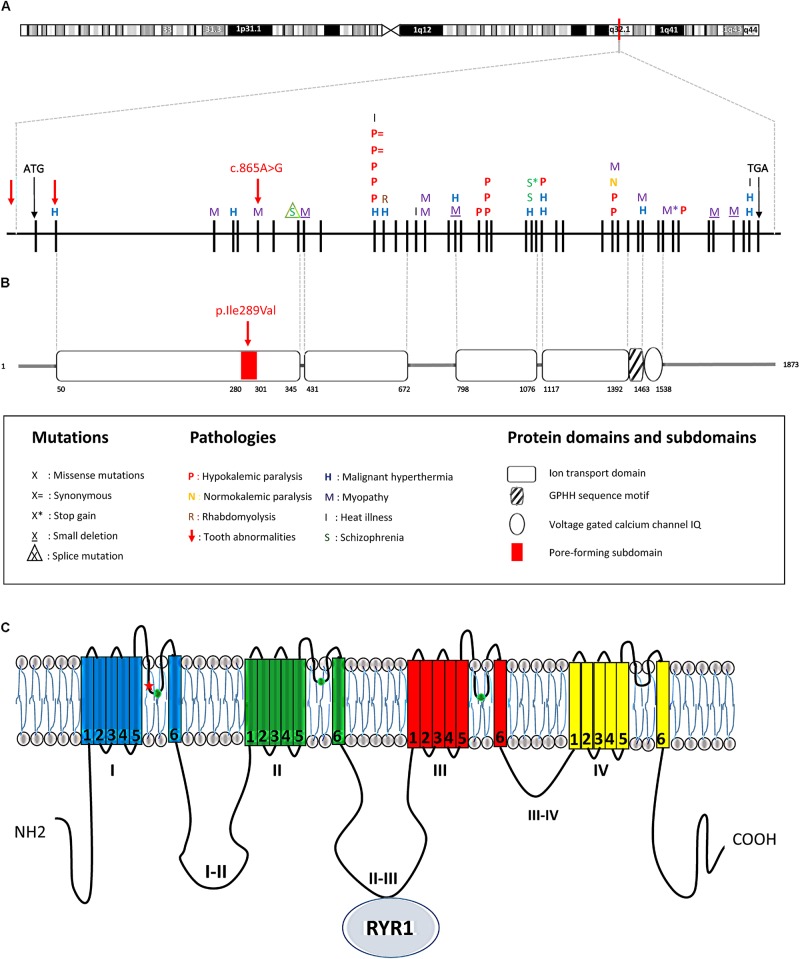
*CACNAS1* mutations and corresponding protein domain. **(A)** The *CACNA1S* human gene is located on chr1q32.1, extends over 73 kb, and contains 44 exons (each vertical black line denotes one exon). The position of the start codon (ATG) is indicated. The mutation detected in this study is highlighted by a red arrow on exon 6 (c.865A > G, p.Ile289Val). All other previously described mutations in the *CACNA1S* gene are symbolized by a single letter above the corresponding exon. **(B)** The corresponding protein domains are represented according to the PFAM database. **(C)** Schematic view of the CACNA1S encoded alpha 1 sub-unit of DHPR. The subunit has a total of four transmembrane domains (I, II, III, IV) composed by six segments (1–6) and three intracellular loop domains (loops I–II, loops II–III, and loops III–IV). The mutated isoleucine (red star) is located in the first pore-forming intramembrane domain close to one of the 3 amino-acids involved in calcium selectivity (green circles). The II_III loop interacts with RYR1 to allow excitation/contraction in muscle.

The UCSC genome browser^5^ was used to model the human *CACNA1S* gene sequence. Previously described mutations extracted from the Human Genome Mutation Database (HGMD^6^) (Stenson et al., 2009) were represented on this gene sequence (**Figure 3**).

The Protein Families (PFAM) database (Punta et al., 2012) was used to search for protein domain organization and the Uniprot database^7^ to collect data for protein subdomains.

### Multiple Protein Sequences Alignment

**FIGURE 4:**
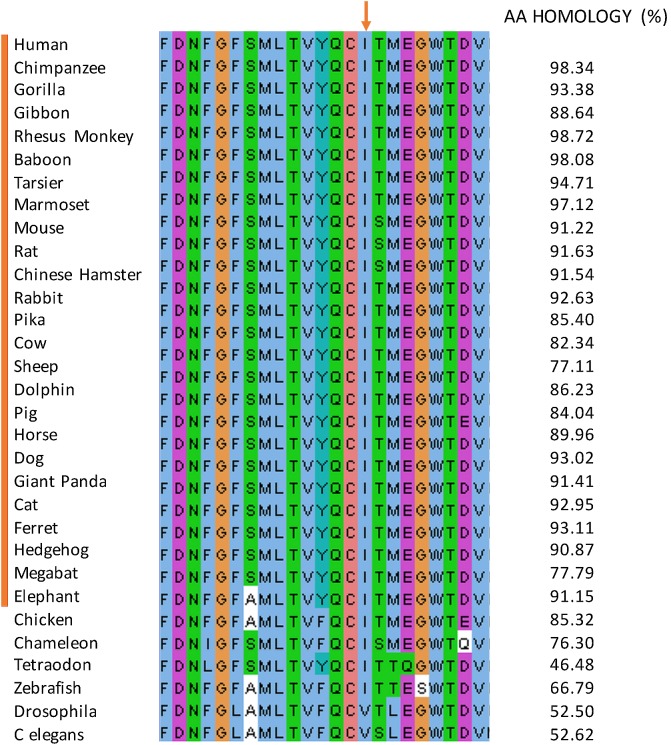
Multiple protein sequence alignment. The sequence alignment of the pore forming subdomain (position 280–301) amongst mammalian 25 species. The alignment is displayed using Jalview and colored according to the ‘ClustalX’ coloring scheme. The isoleucine mutated in the affected individuals (red arrow) is unchanged in all the species.

The CACNA1S Human pore-forming subdomain (280–301) protein sequence was aligned with the CACNA1S sequence of 25 species representative of mammalian lineages and 6 non-mammalian species using Uniprot website^7^. The data were then imported and visualized with Jalview^8^ and colored according to the ‘ClustalX’ coloring scheme (**Figure 4**). The percentage of amino acids identity compared to the Human protein was calculated using the Jalview pairwise alignment.

### Microarray Analysis

In 2013, we developed a transcriptomic atlas (Laugel-Haushalter et al., 2013) that has proven to be a successful tool to search for candidate genes, involved in rare diseases with dental abnormalities, based on their expression in mouse tooth at the E14.5 cap stage. For detailed protocols on microarrays see (Laugel-Haushalter et al., 2013).

## Results

### Clinical Phenotype

Eleven affected individuals from 5 not known to be related non-consanguineous families presented with a unique dental phenotype of disorganized supernumerary cusps pattern. Patients did not suffer from any other medical conditions as assessed by detailed medical history and examination. Patients’ medical records were scrutinized and reviewed a second time after the genotype results to search for specific phenotypes, such as myopathies, and no other existing disease was identified. They were examined clinically for in-depth analysis of their oral morphological changes. Strikingly, all of the affected subjects displayed supernumerary cusps throughout affected molars, [compare **Figure 1** control molar (A, B) with 4/5 cusps versus affected patient with at least 10 extra-cusps (C–F)]. These extra-cusp alterations typically manifested in both maxillary and mandibular molars. The detailed oral phenotype can be seen in patient 1_III.11 (**Supplementary Figures S1A–D**), patient 2_III.7 (**Supplementary Figures S1E–G**), and patient 4_II.3 (**Supplementary Figures S1I–L**). These accessory structures created deep pits and grooves, which resembled “mulberry molars” (Nissanka-Jayasuriya et al., 2016). The location of these supernumerary molar cusps was clearly occlusal and appeared distinct from Carabelli cusps, the latter typically present on the external palatal side of the molar (Hunter et al., 2010) (Carabelli cusp is number 5 in **Figure 1A**). Even in the case of premolars, normally having 2–3 cusps, the unusual presence of multiple accessory cusps with one protruding cusp was clearly visible (see **Figures 1D–F** and **Supplementary Figures S1D,L** for lower premolars). Canines, which typically have a single cusp and a cingulum that could be considered as a small lingual or palatal cusp, also displayed an unusual multicusp pattern around one prominent vestibular cusp (**Supplementary Figures S1K,L**). Other dysmorphic changes in patients extended beyond simple cusp multiplications. About 80% of affected patients’ canine and incisors had exaggerated marginal ridges and unusually large cinguli. Incisor occlusal ridges showed groves or lobes termed “mamelons” (normally present in children’ permanent incisors at tooth eruption and disappearing with wear), that were larger than normal (**Supplementary Figure S1I**). Collectively, we observed widespread changes in the crown cusp pattern and morphology of all teeth in all affected patients.

### Root Changes Accompanying Supernumerary Cusps

Tooth root branching shape anomalies were found in all affected patients and most commonly were root furcation branching deficits. Typically supernumerary cusped molars or premolars also had single tapered roots, instead of normal bifurcated or trifurcated ones found in unaffected individuals (**Figures 1G,H**). Taurodontism, a molar shape anomaly, visible on radiographs, consisting of elongated pulp chambers and apical displacement of the bifurcation or trifurcation of roots is illustrated in **Figure 1H** or in panoramic radiographs of patients from families 1, 2, 3, 4 (**Supplementary Figures S1B,G,H,J**). In all cases both molars and premolars with supernumerary cusps exhibited morphological alterations of root morphology. In the majority of cases (>97%) a single root was found (when normally 2–3 roots on specific premolars or molars). The only teeth that still demonstrated some multiple root formation were the first lower permanent molars (46 or 36; for the tooth numbering system refer to **Supplementary Figure S3**), with an apical displacement of the furcation leading to a taurodontic appearance (**Figure 1H** and **Supplementary Figures S1B,G** blue arrow).

Patient 2_III.7 also had abnormalities of tooth number with tooth agenesis and 2 missing premolars (second lower left 35, upper right 15 premolar). Anomalies of tooth number were also seen in family 3. Patient 3_II.2 had a supernumerary lower right permanent incisor (**Supplementary Figure S1H**).

Individual 4_II.3 (**Supplementary Figures S1I–L**) was the most severely affected, with numerous cusp duplications blocking normal dental occlusion (**Supplementary Figure S1I**).

### Genetic Transmission

Fifteen individuals (11 affected and 4 unaffected) from 5 not known to be related non-consanguineous families were questioned concerning family genetics, then genotyped using salivary samples collected to obtain DNA. In all families, an autosomal dominant mode of inheritance was suspected. **Figure 2** illustrates composite pedigrees/genetic lineages of all affected patients. The dominant inheritance was supported by results showing that no regions of homozygosity were found in affected patients (**Supplementary Table S2**). Moreover, a similar dominant phenotype has been described previously (Skrinjaric et al., 2016).

In the extended pedigree of family 1 with 28 individuals an autosomal dominant pattern of inheritance was clearly visible (**Figure 2**) with affected mother (1_II.10), unaffected father (1_II.11), affected son (1_III.10) and affected daughter (1_III.11). Family 2 comprised 15 individuals amongst which an affected mother (2_II.5), an unaffected father (2_II.6) and both affected children (2_III.6 and 2_III.7). Family 3 comprised two affected females (3_II.1 and 3_II.2). Families 1, 3, 4, 5 affected individuals and especially their parents or grand-parents originated from the same city-Maha Sarakham in the north-eastern region of Thailand. Family 2 was from Roi Et, a city 40 km away from Maha Sarakham.

### Mutation Analysis

To genetically investigate why this rare multicusp, single root pattern might occur, we analyzed the genotype of these patients by WES.

We also analyzed copy number variations using CANOES program (Backenroth et al., 2014) finding no region that was common to all the affected members in a single family.

Sequencing data processing and variant calling (SNV and InDels) revealed from 57,522 to 58,694 genetic variants per proband (**Table 1**). Variant filtering using stringent criteria reduced the number of genetics variants to, respectively, 770, 749, 771, 854, 926, 956, 934, and 798 variants per proband (1_II.11, 1_III.10, 2_II.5, 2_II.6, 2_III.6, 2_III.7, 3_ II.1, 3_ II.2, affected individuals in bold). As the pedigrees suggested an autosomal dominant mode of inheritance with a complete penetrance within a group of patients coming from the same region, we narrowed the selection to heterozygous variants, shared by affected and not present in non-affected individuals, and we were left with only one variant in the *CACNA1S* gene (NM_000069.2: c.865A > G; p.Ile289Val).

**Table 1:** Summary of the WES analysis in families 1–3.

Patient	1_II.11		1_III.1 0		2_II.5		2_II.6		2_III.6		2_III.7		3_II.1		3_II.2	
Type of variant	SNV	Indel	SNV	Indel	SNV	Indel	SNV	Indel	SNV	Indel	SNV	Indel	SNV	Indel	SNV	Indel
Total number of variants	49953	7942	49610	7948	49531	7991	48192	7593	50611	8083	50284	7942	50070	7963	50544	8006
Variants with an allele frequency <1%	1782	590	1732	588	1761	615	1897	628	2065	671	2223	649	2100	626	1880	600
Exclusion of 5′UTR, 3′UTR and intron locations without local splice effect prediction	954	69	917	77	986	79	1076	79	1189	91	1240	75	1145	97	1011	87
Exclusion of synonymous variants without local splice effect prediction	706	64	679	70	696	75	781	73	839	87	887	69	840	94	716	82
Variants consistent with dominant transmission	1 heterozygous variant in *CACNA1S*															

*One heterozygous variant common to all the affected patients (1_III.10, 2_II.5, 2_III.6, 2_III.7, 3_II.1, and 3_II.2) in the genes CACNA1S, was selected for further investigation after exclusion of mutations located in the 5′UTR, 3′UTR, intron, along with synonymous variants without predicted splicing effects*.

To confirm this finding, we subsequently validated familial segregation by Sanger sequencing on all enrolled family members and confirmed that the mutation segregated properly with the disease within all the families (**Figure 2**).

To test for a possible increased presence of the identified variant in the Thai population independent of any pathological context, we sequenced exon 6 of the *CACNA1S* gene for 18 unaffected unrelated control subjects also originating from the north-eastern region of Thailand. Although limited in number, this data confirmed information found in a Thai database^9^ consisting of 32 genotyped individuals and an Asian database (Ngamphiw et al., 2011) of 1719 individuals in which the *CACNA1S* identified variant was never reported. In summary, control subjects never carried this mutation, excluding that this variant was a common polymorphism found in individuals originating from this region (**Supplementary Figure S2**).

The *CACNA1S* gene belongs to a family of genes encoding calcium channels-specifically the pore- forming alpha 1 subunit of the voltage-gated L-type Ca^2+^ channel (dihydropyridine receptor DHPR). It regulates calcium release from the muscle sarcoplasmic reticulum (Schartner et al., 2017). CACNA1S also known as DHPR1, Cav1.1 and CACN1 encodes the 176 kDa subunit of the DHPR channel. This unit is composed of four six-segments (S1–S6) transmembrane domains (forming ion transport domains I, II, III, and IV) and three intracellular loop domains (loops I and II, loops II and III, loops III and IV). The S4 segment of each repeated domain is thought to be the voltage sensor. Intramembrane domains are involved in the pore formation and play a role in the gating and the selectivity of the channel; the II–III loop interacts with Ryanodine receptor 1 (RYR1) to allow excitation/contraction of muscles. The mutation localized to the pore forming subdomain (280–301) of the first ion transport domain (50–345) of the CACNA1S protein (**Figure 3**).

Knowing that all the families shared the same variant and originated from the same region, we checked if the families could be closely related by estimating the kinship coefficient with KING (**Supplementary Table S3**), which suggested that the three families were unrelated. As the families were not closely related, we suspected a founder effect and we decided to look at the rare variants in the *CACNA1S* region. We realized that affected patient were sharing a region with exactly the same SNPs (**Supplementary Table S4**). This could suggest that a portion of chromosome with the mutation was transmitted along generations in a population, confirming a founder effect linked to the mutation and subsequent phenotype.

### Mutations in the Human *CACNA1S* Gene

This mutation occurred at an isoleucine residue that is conserved in the 25 studied and aligned CACNA1S mammalian sequences. Moreover, this isoleucine residue is highly conserved in vertebrates but not in invertebrates (**Figure 4**). The mutation is also predicted to be disease causing by MutationTaster (Schwarz et al., 2014) and possibly damaging by PolyPhen-2 (Polymorphism Phenotyping v2) (PPH2) (Adzhubei et al., 2010). The list of all the bioinformatics algorithms used to determine the pathogenicity of variants and their scores are available for the 3 families in **Supplementary Tables S5**–**S7**). These findings suggest a putative important function for this amino acid.

### Expression of CACNA1S Complex Partners and Interactors in Mouse Tooth Germs

CACNA1S or Cav1.1 is the pore forming subunit of a L-type calcium channel, composed of 4 subunits (CACNA1S, CACNAB1 or CACNAB2, CACNAG1, and CACNA2D1), named the dihydropyridine channel or DHP channel. In myocytes, it regulates calcium release from the sarcoplasmic reticulum by activating the ryanodine receptor (Schartner et al., 2017). To examine a potential role of calcium channels during odontogenesis, we checked for the presence and expression of *Cacna1s* complex partners and known interactors within a E14.5 mouse tooth transcriptomic atlas (Laugel-Haushalter et al., 2013) (**Table 2**).

**Table 2:** Expression of *CACNA1S* complex partners (in bold) and interactors in E14.5 mouse tooth germ.

Gene symbol	Lower molars ± STD	Lower incisors ± STD	Upper molars ± STD	FC lower molars versus lower incisors	*p*-value	FC lower versus upper molars	*p*-value2
**Cacna1s**	7.04 ± 0.06	6.39 ± 0.37	5.92 ± 0.1	1.7402	0.0000582054	1.58492	0.0000703422
**Cacnb1**	8.12 ± 0.09	7.92 ± 0.17	7.78 ± 0.11	1.04509	0.46	1.0344	0.57
**Cacnb2**	7.54 ± 0.11	7.73 ± 0.22	7.64 ± 0.1	−1.16979	0.013	1.12838	0.04
**Cacna2d1**	8.61 ± 0.01	8.91 ± 0.28	8.82 ± 0.08	−1.25004	0.04	−1.05355	0.52
**Cacng1**	7.08 ± 0.07	6.51 ± 0.44	6.01 ± 0.07	1.43416	0.0000630813	1.5501	0.00000326276
Calm1	11.81 ± 0.06	11.72 ± 0.14	11.67 ± 0.03	−1.17861	0.0000945752	1.02461	0.28
Calm2	10.92 ± 0.05	10.91 ± 0.12	10.92 ± 0.11	−1.22554	0.00235493	1.04302	0.41
Calm3	11.01 ± 0.04	10.91 ± 0.21	10.75 ± 0.05	−1.12517	0.01	1.06782	0.09
Dysf	8.25 ± 0.21	8.57 ± 0.03	8.06 ± 0.04	1.08	0.11	1.21	0.00153247
Jsrp1	7.54 ± 0.32	7.94 ± 0.02	7.28 ± 0.05	1.13	0.00534678	1.18	0.000235233
Ryr1	8.06 ± 0.21	7.18 ± 0.57	6.43 ± 0.08	1.58111	0.00279044	1.88642	0.0000587608
Stac	7.97 ± 0.08	7.68 ± 0.12	7.92 ± 0.06	−1.32	0.000197742	1.01	0.87
Stac2	6.42 ± 0.17	6.4 ± 0.08	6.23 ± 0.07	1.02	0.77	1.01	0.96
Stac3	6.63 ± 0.41	7.29 ± 0.12	6.17 ± 0.07	1.71	0.0000202013	1.38	0.000251342

*All the CACNA1S partners and interactors are expressed in all tooth types (lower incisors, lower and upper molars) with the exception of Myh13. Most are more strongly expressed in lower molars.*

We showed that *Cacna1s* and all complex partners were expressed in E14.5 mouse incisors and molars. Indeed, the signal value from genes expressed in the tissue are between 4 and 13, so the genes with a ratio above 4 will be considered as expressed in the tissue. For *Cacna1s* and *Cacnag1*, expression was stronger in lower molars compared to lower incisors and upper molars.

Calmodulin is a calcium sensor known to bind the isoleucine glutamine (IQ) sequence motifs of *CACNA1S* (Halling et al., 2009). We showed that *Calm1*, *Calm2*, and *Calm3* were strongly expressed in all tooth types suggesting that the CACNA1S channel may play a role in calcium sensing and transport in teeth. Ryanodine receptor 1 (RYR1) and STAC proteins have also been described to interact with CACNA1S during muscle contraction (Wong King Yuen et al., 2017). *Ryr1* and *Stac* were strongly expressed in teeth, with a higher expression than *Ryr1*, *Stac2*, and *Stac3* in lower molars. *Dysf* (Dysferlin) and *Jsrp1* (Junctional Sarcoplasmic Reticulum Protein 1) are other known CACNA1S interactors found in E14.5 fetal teeth.

These expression data suggest a possible role of calcium regulated channels directing tissue morphogenic events. The *Cacna1s* gene is indeed expressed during mouse E14.5 cap stage tooth development, consistent with a possible role of this gene in tooth shape patterning and modulation of cusp signaling center activity.

## Discussion

### Previous Reports of CACNA1S-Like Dental Defects

In this paper, we identified mutations in the *CACNA1S* gene as the probable cause of a dental rare disease consisting of major tooth morphogenetic modifications enhancing the number of cusps, altering tooth crown cusp pattern, and inhibiting root branching. (Skrinjaric et al., 2016) described a similar phenotype in three unrelated Croatian families with 17 affected members presenting with cone-shaped premolars, multitubercular molar crowns, pyramidal molar roots with single root canals, shovel-shaped incisors with palatal invaginations, and hypodontia-a condition estimated to be present in less than 1:1,000,000 with an autosomal dominant mode of inheritance. In Ather et al. (2013) microdontia and multicusp patterns were reported, along with tooth discoloration, hypercementosis, and multiple pulp stones suggesting a different disease. Another similar phenotype is lobodontia (Brook and Winder, 1979; Gorlin, 1998; Gorlin et al., 2001; Hennekam et al., 2010; Ather et al., 2013; Kiyan et al., 2013; Skrinjaric et al., 2016), tooth morphology changes somewhat like *Canis lupus familiaris* (dog) or wolf teeth with multiple abnormalities -fanged-like teeth (accentuated cusps), molar multituberculisms, shovel-shaped incisors, hypodontia, and microdontia. In all described cases, potential genetic effectors are to date unknown. Moreover, children born from mothers with syphilis have multicusp mulberry-like molars (Hillson et al., 1998).

### Possible Role of Calcium Signaling in Dental Development

Mutations in the *CACNA1S* gene encoding a calcium ion channel have been a suspected cause of a variety of different diseases. Indeed according to HGMD, 43 mutations involved in seven different diseases have been described in the *CACNA1S* gene (listed in **Supplementary Table S1** and **Figure 3**). These include malignant hyperthermia susceptibility 5, MIM #601887 (Eltit et al., 2012), thyrotoxic periodic paralysis, susceptibility to, 1, MIM #188580 (Kung, 2006), hypokalemic periodic paralysis, type 1, MIM #170400 (Fialho et al., 2018), normokalaemic periodontic paralysis (Fan et al., 2013), myopathy (Schartner et al., 2017), rhabdomyolysis (Vivante et al., 2017), exertional heat illness (Fiszer et al., 2015), and schizophrenia (Fromer et al., 2014; Purcell et al., 2014). Six mutations, localized to the first ion transport domain, were previously reported to result in myopathies, malignant hyperthermia, and schizophrenia. Uniquely, our families had a mutation in the pore-forming subdomain of this first domain (**Figure 3**). Affected individuals did not suffer from any of these conditions. Collectively, our data strongly implicate the observed c.865A > G missense mutation as the most likely cause of the dental phenotype observed in our families.

In all of our patients, the mutation segregates with the phenotype and likely produces defects such as extra cusps, deficient roots, along with other dental alterations. CACNA1S is the pore-forming subunit of a voltage-activated channel, acting as a sensor of depolarization, allowing calcium release and diffusion. In general, channelopathies are diseases resulting from the dysfunction of voltage-gated ion channels, and can induce a number of neurological, muscular, and cardiac defects. Calcium influx and consequent regulation through regulated entry are increasingly recognized as key regulators of various developmental processes (Bates, 2015). To date, no dental malformations have been described in patients with *CACNA1S* mutations. Disruption of other voltage-regulated calcium channels, though, has been shown to alter dentition, produce microdontia or tooth agenesis, dentin formation, tooth eruption defects, or amelogenesis imperfecta [reviewed in Duan (2013)]. For instance, *CACNA1C* disruption in Timothy Syndrome produces small, misplaced teeth due to enamel hypoplasia (Splawski et al., 2004). Roles of ion transporters affecting enamel and dentin biomineralization could stem from calcium roles regulating mineralization, ion homeostasis, pH, or endocytosis. Recently the TRPM4 channel knockdown in dental follicle progenitors was found to cause reduced calcium influx, which actually increases mineralization by reducing dental stem proliferation (Nelson et al., 2013).

Fatemifar et al. (2013) identified through genome wide association study *CACNB2* associated with primary tooth eruption. The voltage-gated calcium channel *CACNA1C* and calcium influx was shown to be involved in regulating mandible development, providing further evidence of a role of these molecules in non-excitable cells during development (Ramachandran et al., 2013).

### Role of the Identified Mutation in *CACNA1S*

We identified a missense mutation in exon 6 of *CACNA1S* (NM_000069.2: c.865A > G; p.Ile289Val). However, we could not exclude that this variant could co-segregate with another non-coding variant. It is likely that this variant produces the dental phenotype we here describe, based on the fact that some association of the *CACNA1S* region with tooth development/anomalies such as with permanent tooth eruption (Geller et al., 2011) and with tooth agenesis (Jonsson et al., 2018) were already reported. The variant was found once among 30974 genomes based on the gnomAD database (Lek et al., 2016) and is present in dbSNP (rs139920212) as supported by several submissions but it could be that the individual dental phenotype was never assessed.

The amino-acid change (p.Ile289Val) is in the intramembranous domain of the protein portion constituting the pore-forming subunit of the voltage-gated calcium channel. Loss of function mutations observed in the *CACNA1S* first domain (p.F275L) and the nearby pore forming subdomain resulted in myopathy (Schartner et al., 2017), a disease that is clearly not present in the affected individuals of our cohort. The mutation that we identified is 3 amino-acids before the amino-acid 292 involved in calcium ion selectivity and permeability. As the mutation is very close to this amino-acid involved in calcium selectivity (**Figure 3**), we propose that changing an isoleucine into a valine could result in a higher accessibility to this amino acid leading to potential alteration of the channel gating or specificity. The mutation that we identified is most probably a gain of function or a modification of the channel permeability possibly allowing other ions to travel through the lumen or increasing the calcium influx. A second review targeting medical assessment found no *CACNA1S* mutation-specific phenotypes (myopathy, for example) in our patients. However, we cannot exclude a possible risk of malignant hyperthermia caused by general anesthesia.

### How Alterations in Ion Gradients Could Alter Cusp Pattern by Placode Signaling Centers

Tooth crown morphology as defined by the number of cusps, their spatial distribution, shape and size is clearly dependent of signaling centers called primary and then secondary enamel knots (Jernvall et al., 1994; Vaahtokari et al., 1996; Thesleff and Jernvall, 1997; Thesleff et al., 2001; Matalova et al., 2005; Harjunmaa et al., 2012). The increase in cusp number does not result from an increase in tooth size, but from an altered primary patterning phase of development (Harjunmaa et al., 2012). Multiple signaling pathways (NF-kappaB, SHH, BMP…) are involved in setting up this complexity which can be increased substantially by adjusting these pathways simultaneously (Harjunmaa et al., 2012).

Teeth are highly evolvable because only small developmental changes are needed to produce large changes in size and number of small cusps (Jernvall, 2000). BMP4 is clearly involved in the induction of the tooth signaling centers (Jernvall et al., 1998).

In mammals, the design that is considered one of the most important characteristics is a three-cusp shape (protocone, paracone, metacone) called a tribosphenic molar. Quadrate (also called quadritubercular) molars have an additional fourth cusp on the lingual side called the hypocone that is present in primates and humans. The modified multiple cusps molar phenotype reported here nevertheless allows the recognition of 3–4 prominent cusps, reminiscent of this primary cusp organization that allowed the evolution and diversity of cusps formation in mammalian species. The complexity and number of cusps may arise by budding of the crown due to the multiplication of signaling centers.

Several mammalian species naturally possess multicuspid incisors, suggesting that mammals have the capacity to form multicuspid teeth regardless of location in the oral jaw. Misregulation of Shh and Bmp signaling in *Lrp4* null mice led to the formation of incisors with molar like cusps (Ohazama et al., 2010). Clearly the multiple cusps pattern encountered in case of *CACNA1S* mutation is not restricted to the molar area.

Ion channels influence developmental processes involving BMP signaling (Bates, 2015). Every cell has a membrane potential set up by the concentration of ions in and out the cell. Ion channels can influence this membrane potential. Voltage gated calcium channels are depolarizing channels making the membrane potential more positive. Ions can travel through the ion channels or gap junctions. BMP signaling pathway and secretion may rely on ion channel function, as demonstrated in hair follicle stem cells and by craniofacial or digit phenotypes observed in Kir2.1 or Cav1.2 dysfunction.

Ion current alterations could change osmotic balance, affecting cell volume, increasing physical stress. Patients with K^+^ channel Kir2.1 mutations can have cleft palate, reduced jaw size, and dental alterations (Yoon et al., 2006), similar to the mouse knockout model implicating its role [(Dahal et al., 2012) and references therein]. Compromised expression of the Drosophila Kir2.1 homolog reduces bone morphogenetic protein BMP signaling, a change that can dramatically alter tooth patterning. Reducing zebrafish BMP levels induce supernumerary cusps and teeth (Jackman et al., 2013). How any ion-induced alterations in dental placode alter tooth development is relatively unexplored. Alterations in dental placode activators (ectodysplasin, activin, and FGF), or inhibitors (BMP or SHH) seem to collectively act to regulate tooth cusp number complexity. Reduced BMP levels acting within the established network of tooth morphogens might increase cusp number or cause other malformations (Harjunmaa et al., 2012). In addition, regulation of intracellular calcium content, through channel-regulated osmotic control might have influenced dental evolution, especially concerning the transition from ocean to land-based species.

It is intriguing, however, why *Cacna1s* transgenic mice were not reported to display a dental phenotype. Knockout-mice with a total loss of function of *Cacna1s* suffered from myopathy mimicking the phenotype observed in Human with an equivalent *CACNA1S* loss of function mutation (Filipova et al., 2018). Mice were severely affected and died by asphyxia at birth but were a good model to study myopathy during embryonic stages. Mice with a mutation in one of the voltage sensor segment R528H (see **Figure 3C**) reproduced the phenotype observed in the Human hypokalemic periodic paralysis and suffered from hindlimb weakness (Wu et al., 2012). Those mouse models confirm that the effects of the mutations (gain or loss of function) are linked to their nature and position in *Cacna1s*. Another mouse model with a mutation in the third pore-forming domain (**Figure 3C**, third intramembrane domain) was also engineered and suffered from fatty acid metabolism alteration (Georgiou et al., 2015). This mutation impacted the channel selectivity and abolished Ca^2+^ binding within *Cacna1s*. Those mice were not investigated for tooth abnormalities so we could not exclude that they could be affected. However, we suggest that the mutation in our patients, localized in the first pore-forming domain, changes the selectivity or the gating of the channel rather than totally abolishing the calcium binding and that this different mechanism results in a different pathophysiology and phenotype.

### Root Patterning

*CACNA1S* mutation also altered tooth root patterning. Tooth crown and root may have different control mechanisms. The formation of the roots and their branching relies on Hertwig’s epithelial root sheath (HERS), which promotes root initiation and elongation through many signaling pathways including BMP (Wang and Feng, 2017). Bmp2-cKO^Sp7-Cre-EGFP^ mouse have short roots (Rakian et al., 2013). However, the mechanism leading to the formation of a single root, or 2 to 3 roots, is unknown. Taurodontism may appear as a failure of early tooth root division leading to an extended pulp chamber along the tooth main axis. This phenotype was recently associated with *Wnt10a* downregulation (Yang et al., 2015). Elevated extracellular calcium was shown to increase BMP2 via L-type Ca^2+^ channel and ERK pathway in human dental pulp cells (Tada et al., 2010). This mechanism may account for the root patterning defect encountered in patient presenting with *CACNA1S* mutation.

This is a first report of the possible involvement of a voltage L-type Ca^2+^ channel, CACNA1S, in tooth crown and root patterning, as discovered by next generation sequencing analysis of families presenting striking multicusp and single root defects. This suggests CACNA1S alterations may produce major dysfunctions in signaling centers shaping odontogenesis.

## Author Contributions

PP and SM collected the salivary samples and detailed the patients’ phenotype. VL-H, CS, VG, JM, AB, and J-FD identified the molecular basis of the disease through NGS assays. VL-H, SM, PP, KN, VG, WP, KC, HD, and AB-Z analyzed the data and wrote the manuscript. PP and AB-Z designed the study and were involved from conception, funding seeking to drafting, and critical review of the manuscript. All authors therefore contributed to conception, design, data acquisition, analysis and interpretation, drafted, and critically revised the manuscript. All authors gave final approval and agreed to be accountable for all aspects of the work.

## Conflict of Interest Statement

The authors declare that the research was conducted in the absence of any commercial or financial relationships that could be construed as a potential conflict of interest.

## Footnotes

1 http://www.phenodent.org

2 www.dnagenotek.com

3 http://www4a.biotec.or.th/thaisnp2/

4 http://evs.gs.washington.edu/EVS/

5 http://genome-euro.ucsc.edu

6 http://www.hgmd.cf.ac.uk

7 http://www.uniprot.org

8 http://www.jalview.org/

9 http://www4a.biotec.or.th/thaisnp2/

## Supplementary Material

FIGURE S1 Tooth cusp patterning defects found in affected families. The intraoral photographs and panoramic radiograph of affected daughter from family 1 (1_III.11) showed tooth morphology alteration especially multiple accessory cusps in both premolars **(A–D)** or upper **(C)** and lower **(D)** molars. Panoramic radiograph **(B)** showed root branching anomalies, with single roots present for all teeth except the first lower permanent molars presenting with a taurodontism-like phenotype **(B)**. Lateral upper incisors **(A,C)** and multicusp premolars were microdont **(C)**. In the lower arch a similar cusp pattern alteration was visible but each lower premolar showed a prominent single protruding cusp **(D)**. The affected son from family 2 (2_III.7) showed the same tooth dysmorphology phenotype **(E,F)**. The panoramic radiograph from this person showed the similar root alterations **(G)**, the blue arrow pointed toward taurodontic lower right first permanent molar (46). The contralateral tooth (36) had the same taurodontic dysmorphic appearance. In addition, two premolars were missing in both the maxilla and the mandible (red ^∗^). Tooth morphology defects and single root pattern were also observed on the panoramic radiograph from the female (3_II.2) from family 3 **(H)**. A supernumerary lower incisor was present **(H)**. Family 4 affected son (4_II.3) showed the most severe phenotype **(I–L)**. The occlusal ridge of the lower incisors exhibited “mamelons” or indentations. Both upper and lower canines showed multiple cusps instead of their tapered shape **(K,L)**. Multiple cusps interfered with the occlusion **(I)**.

FIGURE S2 Sanger sequencing of *CACNA1S* exon 6 from 18 unaffected and unrelated Thai-patient controls originating from the same region as our index cases. None of the control samples presented the mutation, excluding a SNPs carried by individuals from this region.

FIGURE S3 Human permanent teeth nomenclature. Numerotation designing human permanent teeth according to their type and location following the FDI two digits (No Authors, 1988) and ISO 3950:2016 (*Dentistry — Designation system for teeth and areas of the oral cavity*) recommendations. Ca, Canines; PreM, Premolars. The image is extracted from the D[4]/phenodent Diagnosing Dental Defect Database (www.phenodent.org).

TABLE S1 *CACNA1S* mutations and associated pathologies reported in the literature. The 43 *CACNA1S* known mutations according to HGMD are shown in this table.

TABLE S2 Homozygosity regions detected in families 1–3. Homozygosity regions for the nine individuals analyzed by whole exome sequencing (1_II.11, 1_III.10, 2_II.5, 2_II.6, 2_III.6, 2_III.7, 3_II.1, 3_II.2) were reported in this table. No homozygosity region was common to all affected individuals (1_III.10, 2_II.5, 2_III.6, 2_III.7, 3_II.1, 3_II.2) suggesting that the phenotype did not arise because of shared homozygous mutation.

TABLE S3 Estimation of family relationship inference for family 1, 2, and 3 by KING program. N_SNP: The number of SNPs that do not have missing genotypes in either of the individual, HetHet: Proportion of SNPs with double heterozygotes (e.g., AG and AG), IBS0: Proportion of SNPs with zero IBS (identical-by-state) (e.g., AA and GG), Kinship: Estimated kinship coefficient from the SNP data.

TABLE S4 Common rare variants shared by affected individuals in the CACNA1S region. Both alleles of each individual are represented, the blue region on one allele of each affected individual corresponds to the region with common rare variants shared by all affected individuals. Affected individuals are in bold. The variant corresponding to the *CACNA1S* mutation is in red.

TABLE S5 Variant pathogenicity predictions in family 1. This table summarizes the scores of the bioinformatics algorithms used to predict the pathogenicity of variants. The columns were annotated using the Varank software (Geoffroy et al., 2015).

TABLE S6 Variant pathogenicity predictions in family 2. This table summarizes the scores of the bioinformatics algorithms used to predict the pathogenicity of variants. The columns were annotated using the Varank software (Geoffroy et al., 2015).

TABLE S7 Variant pathogenicity predictions in family 3. This table summarizes the scores of the bioinformatics algorithms used to predict the pathogenicity of variants. The columns were annotated using the Varank software (Geoffroy et al., 2015).
