# Supplementary figures and images for: Genetic Evidence Supporting the Role of the Calcium Channel, CACNA1S, in Tooth Cusp and Root Patterning
Source: Front Physiol. 2018 Sep 26;9:1329. doi: 10.3389/fphys.2018.01329 (PMC6170876; doi:10.3389/fphys.2018.01329)

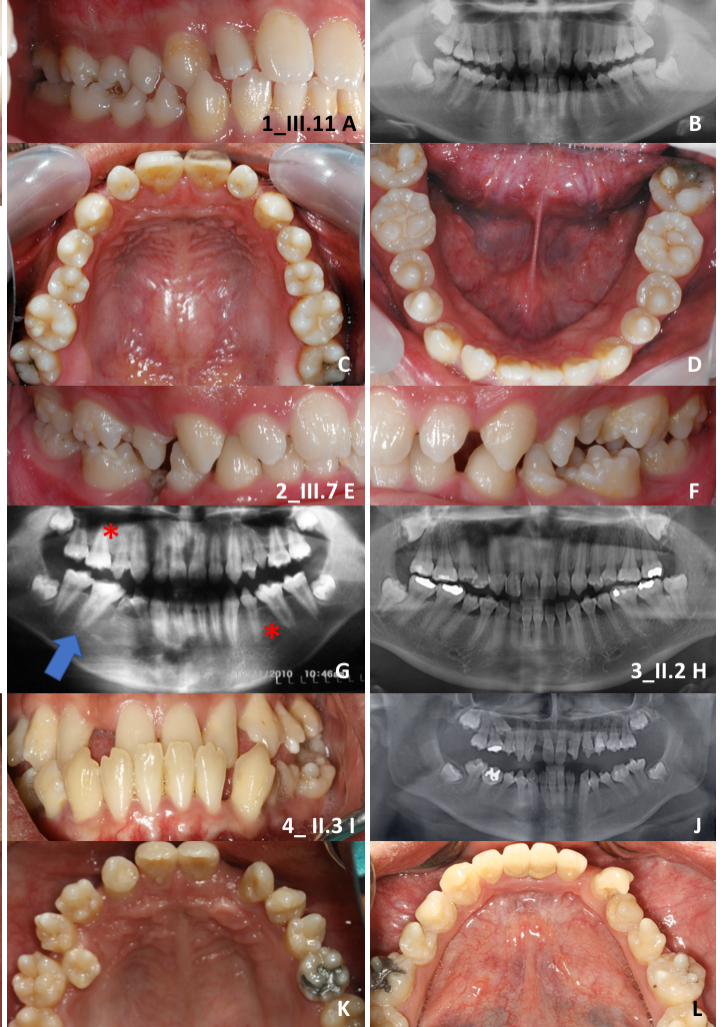

Supplement: FIGURE S1 — Tooth cusp patterning defects found in affected families. The intraoral photographs and panoramic radiograph of affected daughter from family 1 (1_III.11) showed tooth morphology alteration especially multiple accessory cusps in both premolars (A–D) or upper (C) and lower (D) molars. Panoramic radiograph (B) showed root branching anomalies, with single roots present for all teeth except the first lower permanent molars presenting with a taurodontism-like phenotype (B). Lateral upper incisors (A,C) and multicusp premolars were microdont (C). In the lower arch a similar cusp pattern alteration was visible but each lower premolar showed a prominent single protruding cusp (D). The affected son from family 2 (2_III.7) showed the same tooth dysmorphology phenotype (E,F). The panoramic radiograph from this person showed the similar root alterations (G), the blue arrow pointed toward taurodontic lower right first permanent molar (46). The contralateral tooth (36) had the same taurodontic dysmorphic appearance. In addition, two premolars were missing in both the maxilla and the mandible (red ∗). Tooth morphology defects and single root pattern were also observed on the panoramic radiograph from the female (3_II.2) from family 3 (H). A supernumerary lower incisor was present (H). Family 4 affected son (4_II.3) showed the most severe phenotype (I–L). The occlusal ridge of the lower incisors exhibited “mamelons” or indentations. Both upper and lower canines showed multiple cusps instead of their tapered shape (K,L). Multiple cusps interfered with the occlusion (I). [file Image_1.TIFF]

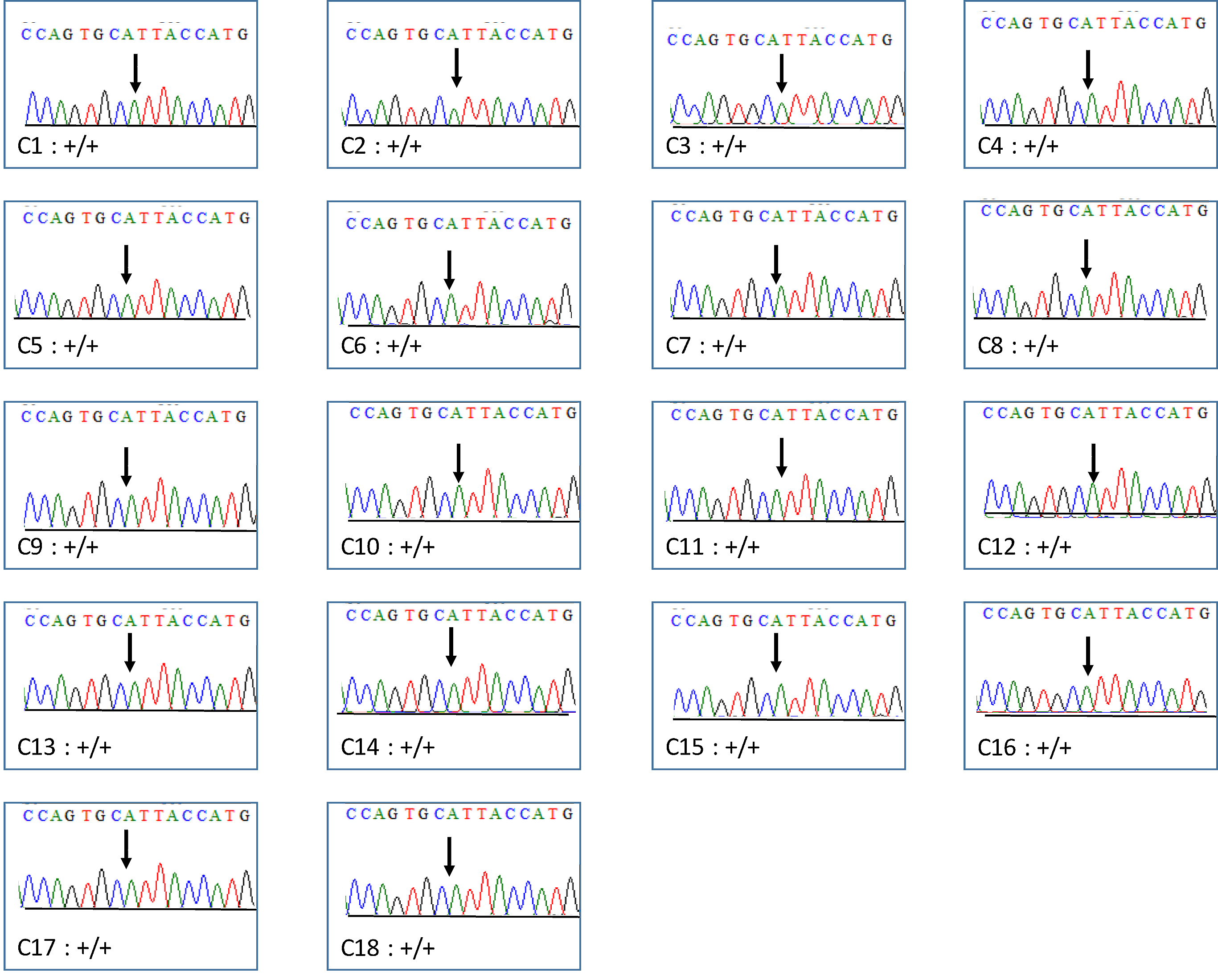

Supplement: FIGURE S2 — Sanger sequencing of CACNA1S exon 6 from 18 unaffected and unrelated Thai-patient controls originating from the same region as our index cases. None of the control samples presented the mutation, excluding a SNPs carried by individuals from this region. [file Image_2.JPEG]

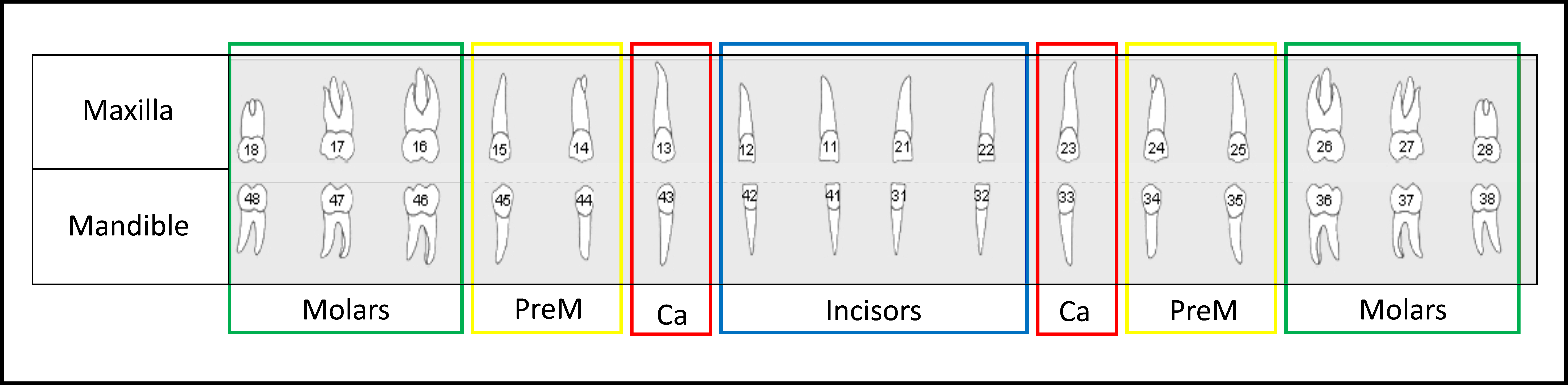

Supplement: FIGURE S3 — Human permanent teeth nomenclature. Numerotation designing human permanent teeth according to their type and location following the FDI two digits (No Authors, 1988) and ISO 3950:2016 (Dentistry — Designation system for teeth and areas of the oral cavity) recommendations. Ca, Canines; PreM, Premolars. The image is extracted from the D[4]/phenodent Diagnosing Dental Defect Database (www.phenodent.org). [file Image_3.JPEG]
