# Supplementary material for: Genetic Evidence Supporting the Role of the Calcium Channel, CACNA1S, in Tooth Cusp and Root Patterning
Source: Front Physiol. 2018 Sep 26;9:1329. doi: 10.3389/fphys.2018.01329 (PMC6170876; doi:10.3389/fphys.2018.01329)
Supplement: TABLE S1 — CACNA1S mutations and associated pathologies reported in the literature. The 43 CACNA1S known mutations according to HGMD are shown in this table. [file Table_1.DOCX]

| **Missense mutation** | **Pathology** | **References** |
| --- | --- | --- |
| c.206C>G;p.A69G | Malignant hyperthermia | (Levano et al., 2017) |
| c.298G>A;p.E100K | Myopathy | (Schartner et al., 2017) |
| c.520C>T;p.R174W | Malignant hyperthermia | (Bannister and Beam, 2013; Carpenter et al., 2009; Eltit et al., 2012) |
| c.825C>A;p.F275L | Myopathy | (Schartner et al., 2017) |
| c.1493G>A;p.R498H | Exertional heat illness | (Fiszer et al., 2015) |
| c.1493G>T;p.R498L | Malignant hyperthermia | (Gillies et al., 2015) |
| c.1551C>T;p.G517= | Hypokalaemic periodic paralysis, association with | (Dias da Silva et al., 2002) |
| c.1564C>T;p.L522= | Hypokalaemic periodic paralysis, association with | (Dias da Silva et al., 2002) |
| c.1582C>T;p.R528C | Hypokalaemic periodic paralysis | (Yang et al., 2014) |
| c.1582C>G;p.R528G | Hypokalaemic periodic paralysis | (Kil and Kim, 2010; Wang et al., 2005) |
| c.1583G>A;p.R528H | Hypokalaemic periodic paralysis | (Domínguez-Morán et al., 2000; Elbaz et al., 1995; Jurkat-Rott et al., 1994) |
| c.1678G>T;p.A560S | Rhabdomyolysis | (Vivante et al., 2017) |
| c.1817G>A;p.S606N | Malignant hyperthermia | (Fiszer et al., 2015; Maxwell et al., 2016) |
| c.2047C>T;p.R683C | Exertional heat illness | (Fiszer et al., 2015) |
| c.2225C>A;p.P742Q | Myopathy | (Schartner et al., 2017) |
| c.2224C>T;p.P742S | Myopathy | (Schartner et al., 2017) |
| c.2440G>A;p.A814T | Malignant hyperthermia | (Gillies et al., 2015) |
| c.2627T>A;p.V876E | Hypokalaemic periodic paralysis | (Ke et al., 2009; Yang et al., 2015) |
| c.2691G>T;p.R897S | Hypokalaemic periodic paralysis | (Chabrier et al., 2008; Hanchard et al., 2013) |
| c.2698A>G;p.R900G | Hypokalaemic periodic paralysis | (Hirano et al., 2011) |
| **c.2700G>T;p.R900S** | **Hypokalaemic periodic paralysis** | (Ke et al., 2015; Matthews et al., 2009) |
| c.2936A>G;p.D979G | Schizophrenia | (Fromer et al., 2014) |
| c.3026C>A;p.T1009K | Malignant hyperthermia | (Fiszer et al., 2015; Kim et al., 2013) |
| c.3048G>A;p.W1016* | Schizophrenia | (Purcell et al., 2014) |
| c.3256C>T;p.R1086C | Hypokalaemic periodic paralysis | (Amendola et al., 2015; Jurkat-Rott et al., 2000) |
| c.3257G>A;p.R1086H | Malignant hyperthermia | (Monnier et al., 1997) |
| c.3256C>A;p.R1086S | Malignant hyperthermia | (Olfson et al., 2015; Toppin et al., 2010) |
| (Kim et al., 2005, 2011)c.3715C>G;p.R1239G | Hypokalaemic periodic paralysis | (Kim et al., 2005, 2011) |
| c.3716G>A;p.R1239H | Hypokalaemic periodic paralysis | (Elbaz et al., 1995; Houinato et al., 2007; Jurkat-Rott et al., 2009) |
| c.3724A>G;p.R1242G | Normokalaemic periodic paralysis | (Fan et al., 2013) |
| c.3795G>T;p.Q1265H | Myopathy | (Hunter et al., 2015; Schartner et al., 2017) |
| c.4060A>T;p.T1354S | Malignant hyperthermia | (Amendola et al., 2015; Dorschner et al., 2013; Pirone et al., 2010) |
| c.4099C>G;p.L1367V | Myopathy | (Schartner et al., 2017) |
| c.4453C>T;p.Q1485* | Myopathy | (Schartner et al., 2017) |
| c.4639C>T;p.R1547W | Hypokalaemic periodic paralysis with malignant hyperthermia susceptibility 5 | (Tian et al., 2015) |
| c.5515C>T;p.P1839S | Exertional heat illness | (Fiszer et al., 2015; Maxwell et al., 2016) |
| c.5550C>A;p.N1850K | Malignant hyperthermia | (Levano et al., 2017) |
| c.5570G>A;p.S1857N | Malignant hyperthermia | (Levano et al., 2017) |
| **Splice mutation** | **Pathology** | **References** |
| c.1004+2T>C | Schizophrenia | (Purcell et al., 2014) |
| **Small deletions** | **Pathology** | **References** |
| c.1189_1190delAG | Myopathy | (Schartner et al., 2017) |
| c.2371delC | Myopathy | (Schartner et al., 2017) |
| c.4947delA | Myopathy | (Hunter et al., 2015) |
| c.4967delT | Myopathy | (Schartner et al., 2017) |

Amendola, L. M., Dorschner, M. O., Robertson, P. D., Salama, J. S., Hart, R., Shirts, B. H., et al. (2015). Actionable exomic incidental findings in 6503 participants: challenges of variant classification. *Genome Res.* 25, 305–315. doi:10.1101/gr.183483.114.

Bannister, R. A., and Beam, K. G. (2013). Impaired gating of an L-Type Ca(2+) channel carrying a mutation linked to malignant hyperthermia. *Biophys. J.* 104, 1917–1922. doi:10.1016/j.bpj.2013.03.035.

Carpenter, D., Ringrose, C., Leo, V., Morris, A., Robinson, R. L., Halsall, P. J., et al. (2009). The role of CACNA1S in predisposition to malignant hyperthermia. *BMC Med. Genet.* 10, 104. doi:10.1186/1471-2350-10-104.

Chabrier, S., Monnier, N., and Lunardi, J. (2008). Early onset of hypokalaemic periodic paralysis caused by a novel mutation of the CACNA1S gene. *J. Med. Genet.* 45, 686–688. doi:10.1136/jmg.2008.059766.

Dias da Silva, M. R., Cerutti, J. M., Tengan, C. H., Furuzawa, G. K., Vieira, T. C. A., Gabbai, A. A., et al. (2002). Mutations linked to familial hypokalaemic periodic paralysis in the calcium channel alpha1 subunit gene (Cav1.1) are not associated with thyrotoxic hypokalaemic periodic paralysis. *Clin. Endocrinol. (Oxf.)* 56, 367–375.

Domínguez-Morán, J. A., Barón, M., de Blas, G., Orensanz, L. M., and Jiménez-Escrig, A. (2000). Clinical-molecular study of a family with essential tremor, late onset seizures and periodic paralysis. *Seizure* 9, 493–497. doi:10.1053/seiz.2000.0454.

Dorschner, M. O., Amendola, L. M., Turner, E. H., Robertson, P. D., Shirts, B. H., Gallego, C. J., et al. (2013). Actionable, pathogenic incidental findings in 1,000 participants’ exomes. *Am. J. Hum. Genet.* 93, 631–640. doi:10.1016/j.ajhg.2013.08.006.

Elbaz, A., Vale-Santos, J., Jurkat-Rott, K., Lapie, P., Ophoff, R. A., Bady, B., et al. (1995). Hypokalemic periodic paralysis and the dihydropyridine receptor (CACNL1A3): genotype/phenotype correlations for two predominant mutations and evidence for the absence of a founder effect in 16 caucasian families. *Am. J. Hum. Genet.* 56, 374–380.

Eltit, J. M., Bannister, R. A., Moua, O., Altamirano, F., Hopkins, P. M., Pessah, I. N., et al. (2012). Malignant hyperthermia susceptibility arising from altered resting coupling between the skeletal muscle L-type Ca2+ channel and the type 1 ryanodine receptor. *Proc. Natl. Acad. Sci. U. S. A.* 109, 7923–7928. doi:10.1073/pnas.1119207109.

Fan, C., Lehmann-Horn, F., Weber, M.-A., Bednarz, M., Groome, J. R., Jonsson, M. K. B., et al. (2013). Transient compartment-like syndrome and normokalaemic periodic paralysis due to a Ca(v)1.1 mutation. *Brain J. Neurol.* 136, 3775–3786. doi:10.1093/brain/awt300.

Fiszer, D., Shaw, M.-A., Fisher, N. A., Carr, I. M., Gupta, P. K., Watkins, E. J., et al. (2015). Next-generation Sequencing of RYR1 and CACNA1S in Malignant Hyperthermia and Exertional Heat Illness. *Anesthesiology* 122, 1033–1046. doi:10.1097/ALN.0000000000000610.

Fromer, M., Pocklington, A. J., Kavanagh, D. H., Williams, H. J., Dwyer, S., Gormley, P., et al. (2014). De novo mutations in schizophrenia implicate synaptic networks. *Nature* 506, 179–184. doi:10.1038/nature12929.

Gillies, R. L., Bjorksten, A. R., Du Sart, D., and Hockey, B. M. (2015). Analysis of the entire ryanodine receptor type 1 and alpha 1 subunit of the dihydropyridine receptor (CACNA1S) coding regions for variants associated with malignant hyperthermia in Australian families. *Anaesth. Intensive Care* 43, 157–166.

Goodloe, A. H., Evans, J. M., Middha, S., Prasad, A., and Olson, T. M. (2014). Characterizing genetic variation of adrenergic signalling pathways in Takotsubo (stress) cardiomyopathy exomes. *Eur. J. Heart Fail.* 16, 942–949. doi:10.1002/ejhf.145.

Hanchard, N. A., Murdock, D. R., Magoulas, P. L., Bainbridge, M., Muzny, D., Wu, Y., et al. (2013). Exploring the utility of whole-exome sequencing as a diagnostic tool in a child with atypical episodic muscle weakness. *Clin. Genet.* 83, 457–461. doi:10.1111/j.1399-0004.2012.01951.x.

Hirano, M., Kokunai, Y., Nagai, A., Nakamura, Y., Saigoh, K., Kusunoki, S., et al. (2011). A novel mutation in the calcium channel gene in a family with hypokalemic periodic paralysis. *J. Neurol. Sci.* 309, 9–11. doi:10.1016/j.jns.2011.07.046.

Houinato, D., Laleye, A., Adjien, C., Adjagba, M., Sternberg, D., Hilbert, P., et al. (2007). Hypokalaemic periodic paralysis due to the CACNA1S R1239H mutation in a large African family. *Neuromuscul. Disord. NMD* 17, 419–422. doi:10.1016/j.nmd.2007.01.020.

Hunter, J. M., Ahearn, M. E., Balak, C. D., Liang, W. S., Kurdoglu, A., Corneveaux, J. J., et al. (2015). Novel pathogenic variants and genes for myopathies identified by whole exome sequencing. *Mol. Genet. Genomic Med.* 3, 283–301. doi:10.1002/mgg3.142.

Jurkat-Rott, K., Lehmann-Horn, F., Elbaz, A., Heine, R., Gregg, R. G., Hogan, K., et al. (1994). A calcium channel mutation causing hypokalemic periodic paralysis. *Hum. Mol. Genet.* 3, 1415–1419.

Jurkat-Rott, K., McCarthy, T., and Lehmann-Horn, F. (2000). Genetics and pathogenesis of malignant hyperthermia. *Muscle Nerve* 23, 4–17.

Jurkat-Rott, K., Weber, M.-A., Fauler, M., Guo, X.-H., Holzherr, B. D., Paczulla, A., et al. (2009). K+-dependent paradoxical membrane depolarization and Na+ overload, major and reversible contributors to weakness by ion channel leaks. *Proc. Natl. Acad. Sci. U. S. A.* 106, 4036–4041. doi:10.1073/pnas.0811277106.

Ke, Q., He, F., Lu, L., Yu, P., Jiang, Y., Weng, C., et al. (2015). The R900S mutation in CACNA1S associated with hypokalemic periodic paralysis. *Neuromuscul. Disord. NMD* 25, 955–958. doi:10.1016/j.nmd.2015.09.006.

Ke, T., Gomez, C. R., Mateus, H. E., Castano, J. A., and Wang, Q. K. (2009). Novel CACNA1S mutation causes autosomal dominant hypokalemic periodic paralysis in a South American family. *J. Hum. Genet.* 54, 660–664. doi:10.1038/jhg.2009.92.

Kil, T.-H., and Kim, J.-B. (2010). Severe respiratory phenotype caused by a de novo Arg528Gly mutation in the CACNA1S gene in a patient with hypokalemic periodic paralysis. *Eur. J. Paediatr. Neurol. EJPN Off. J. Eur. Paediatr. Neurol. Soc.* 14, 278–281. doi:10.1016/j.ejpn.2009.08.004.

Kim, J. B., Lee, K. Y., and Hur, J. K. (2005). A Korean family of hypokalemic periodic paralysis with mutation in a voltage-gated calcium channel (R1239G). *J. Korean Med. Sci.* 20, 162–165. doi:10.3346/jkms.2005.20.1.162.

Kim, J. H., Jarvik, G. P., Browning, B. L., Rajagopalan, R., Gordon, A. S., Rieder, M. J., et al. (2013). Exome sequencing reveals novel rare variants in the ryanodine receptor and calcium channel genes in malignant hyperthermia families. *Anesthesiology* 119, 1054–1065. doi:10.1097/ALN.0b013e3182a8a998.

Kim, J.-B., Lee, G.-M., Kim, S.-J., Yoon, D.-H., and Lee, Y.-H. (2011). Expression patterns of two potassium channel genes in skeletal muscle cells of patients with familial hypokalemic periodic paralysis. *Neurol. India* 59, 527–531. doi:10.4103/0028-3886.84331.

Levano, S., Gonzalez, A., Singer, M., Demougin, P., Rüffert, H., Urwyler, A., et al. (2017). Resequencing array for gene variant detection in malignant hyperthermia and butyrylcholinestherase deficiency. *Neuromuscul. Disord. NMD* 27, 492–499. doi:10.1016/j.nmd.2017.02.008.

Matthews, E., Labrum, R., Sweeney, M. G., Sud, R., Haworth, A., Chinnery, P. F., et al. (2009). Voltage sensor charge loss accounts for most cases of hypokalemic periodic paralysis. *Neurology* 72, 1544–1547. doi:10.1212/01.wnl.0000342387.65477.46.

Maxwell, K. N., Hart, S. N., Vijai, J., Schrader, K. A., Slavin, T. P., Thomas, T., et al. (2016). Evaluation of ACMG-Guideline-Based Variant Classification of Cancer Susceptibility and Non-Cancer-Associated Genes in Families Affected by Breast Cancer. *Am. J. Hum. Genet.* 98, 801–817. doi:10.1016/j.ajhg.2016.02.024.

Monnier, N., Procaccio, V., Stieglitz, P., and Lunardi, J. (1997). Malignant-hyperthermia susceptibility is associated with a mutation of the alpha 1-subunit of the human dihydropyridine-sensitive L-type voltage-dependent calcium-channel receptor in skeletal muscle. *Am. J. Hum. Genet.* 60, 1316–1325.

Olfson, E., Cottrell, C. E., Davidson, N. O., Gurnett, C. A., Heusel, J. W., Stitziel, N. O., et al. (2015). Identification of Medically Actionable Secondary Findings in the 1000 Genomes. *PloS One* 10, e0135193. doi:10.1371/journal.pone.0135193.

Pirone, A., Schredelseker, J., Tuluc, P., Gravino, E., Fortunato, G., Flucher, B. E., et al. (2010). Identification and functional characterization of malignant hyperthermia mutation T1354S in the outer pore of the Cavalpha1S-subunit. *Am. J. Physiol. Cell Physiol.* 299, C1345-1354. doi:10.1152/ajpcell.00008.2010.

Purcell, S. M., Moran, J. L., Fromer, M., Ruderfer, D., Solovieff, N., Roussos, P., et al. (2014). A polygenic burden of rare disruptive mutations in schizophrenia. *Nature* 506, 185–190. doi:10.1038/nature12975.

Schartner, V., Romero, N. B., Donkervoort, S., Treves, S., Munot, P., Pierson, T. M., et al. (2017). Dihydropyridine receptor (DHPR, CACNA1S) congenital myopathy. *Acta Neuropathol. (Berl.)* 133, 517–533. doi:10.1007/s00401-016-1656-8.

Stenson, P. D., Mort, M., Ball, E. V., Howells, K., Phillips, A. D., Thomas, N. S., et al. (2009). The Human Gene Mutation Database: 2008 update. *Genome Med.* 1, 13. doi:10.1186/gm13.

Tian, X., Liang, W.-C., Feng, Y., Wang, J., Zhang, V. W., Chou, C.-H., et al. (2015). Expanding genotype/phenotype of neuromuscular diseases by comprehensive target capture/NGS. *Neurol. Genet.* 1, e14. doi:10.1212/NXG.0000000000000015.

Toppin, P. J., Chandy, T. T., Ghanekar, A., Kraeva, N., Beattie, W. S., and Riazi, S. (2010). A report of fulminant malignant hyperthermia in a patient with a novel mutation of the CACNA1S gene. *Can. J. Anaesth. J. Can. Anesth.* 57, 689–693. doi:10.1007/s12630-010-9314-4.

Vivante, A., Ityel, H., Pode-Shakked, B., Chen, J., Shril, S., van der Ven, A. T., et al. (2017). Exome sequencing in Jewish and Arab patients with rhabdomyolysis reveals single-gene etiology in 43% of cases. *Pediatr. Nephrol. Berl. Ger.* 32, 2273–2282. doi:10.1007/s00467-017-3755-8.

Wang, Q., Liu, M., Xu, C., Tang, Z., Liao, Y., Du, R., et al. (2005). Novel CACNA1S mutation causes autosomal dominant hypokalemic periodic paralysis in a Chinese family. *J. Mol. Med. Berl. Ger.* 83, 203–208. doi:10.1007/s00109-005-0638-4.

Yang, B., Yang, Y., Tu, W., Shen, Y., and Dong, Q. (2014). A rare case of unilateral adrenal hyperplasia accompanied by hypokalaemic periodic paralysis caused by a novel dominant mutation in CACNA1S: features and prognosis after adrenalectomy. *BMC Urol.* 14, 96. doi:10.1186/1471-2490-14-96.

Yang, H., Zhang, H., and Xing, X. (2015). V876E mutation in CACNA1S gene associated with severe hypokalemic periodic paralysis in a Chinese woman. *J. Formos. Med. Assoc. Taiwan Yi Zhi* 114, 377–378. doi:10.1016/j.jfma.2013.07.007.
